# Multi-Objective Optimization of Injection Molding Process Parameters for Junction Boxes Based on BP Neural Network and NSGA-II Algorithm

**DOI:** 10.3390/ma18030577

**Published:** 2025-01-27

**Authors:** Tengjiao Hong, Dong Huang, Fengjuan Ding, Liyong Zhang, Fulong Dong, Lei Chen

**Affiliations:** 1 College of Intelligent Manufacturing, Anhui Science and Technology University, Chuzhou 233100, China; hongtengjiao@ahstu.edu.cn (T.H.); hdong12@126.com (D.H.); zhangly@ahstu.edu.cn (L.Z.); dongfl@ahstu.edu.cn (F.D.); 2 School of Business Administration, Stamford International University, Bangkok 10250, Thailand; 3 Fengyang County Science and Technology Innovation Service Center, Chuzhou 233100, China; lchen2010@126.com

**Keywords:** junction box, warping deformation, volume shrinkage rate, BP neural network, multi-objective optimization, process parameters

## Abstract

Many factors affect the quality of the injection molding of plastic products, including the process parameters, mold materials, type and geometry of plastic parts, cooling system, pouring system, etc. A multi-objective optimization method for injection molding process parameters based on the BP neural network and NSGA-II algorithm is proposed to address the problem of product quality defects caused by unreasonable process parameter settings. Taking the junction box shell as the object, numerical simulation was carried out using Moldflow2019 software and a six-factor five-level orthogonal experiment was designed to explore the influence of injection molding process parameters, such as the mold temperature, melt temperature, injection pressure, holding pressure, holding time, and cooling time, on the volume shrinkage rate and warpage deformation of the junction box. Based on a numerical simulation, the BP neural network and NSGA-II algorithm were used to optimize the optimal combination of injection molding process parameters, volume shrinkage rate, and warpage deformation. The research results indicate that the melt temperature has the most significant impact on the quality of the injection molding of junction boxes, followed by the holding time, holding pressure, cooling time, injection pressure, and mold temperature. After optimization using the BP neural network and the NSGA-II algorithm, the optimal process parameter combination was obtained with a melt temperature of 230.03 °C, a mold temperature of 51.27 °C, an injection pressure of 49.13 MPa, a holding pressure of 69.01 MPa, a holding time of 15.48 s, and a cooling time of 34.91 s. At this time, the volume shrinkage rate and warpage deformation of the junction box were 6.905% and 0.991 mm, respectively, which decreased by 33.2% and 3.8% compared to the average volume shrinkage rate (10.34884%) and warpage deformation (1.030764 mm) before optimization. The optimization effect was significant. In addition, the errors between the volume shrinkage rate and warpage deformation predicted by BP-NSGA-II and the simulated values using Moldflow software were 1.9% and 3.4%, respectively, indicating that the optimization method based on the BP neural network model and NSGA-II algorithm is reliable.

## 1. Introduction

Plastic has the advantages of stable performance and low density and is widely used in the information industry, aerospace, transportation, and other fields of the national economy [1]. It has become one of the important materials supporting the development of China’s national economy and meeting people’s daily life needs. Plastic Injection molding is widely used in the manufacturing of various plastic products due to its high production efficiency, low cost, and good product flexibility [2]. Plastic injection molding is a complex linear process with multiple parameters and interactions, which requires high requirements for process parameter settings [3,4]. Similar to traditional metal processing, plastic-injection-molded products also have defects, such as uneven shrinkage, weld lines, and warping deformation [5]. At present, mold manufacturers mostly obtain qualified plastic products by repeatedly repairing and modifying molds to address the problem of plastic product warping and deformation. This method of improving the quality of injection molded parts not only increases production costs but also takes a considerable amount of time, attention, and resources. With the surge in the demand for plastic products and rapid advances in the plastic industry, reducing the warping and deformation of plastic products during production has become an urgent need to be addressed in the injection mold industry.

Domestic and foreign scholars have conducted extensive research on plastic part warping deformation and have made significant progress. Li et al. [6] used Moldflow software and orthogonal experimental methods to study the effect of process parameters on the shrinkage rate of PFA (perfluoroalkoxy alkane) parts. The research results showed that the injection rate had the most significant impact on the shrinkage of the pipe length. In contrast, the melt temperature, holding pressure, and screw speed had the greatest impact on the shrinkage of the pipe’s outer diameter. Guo et al. [7] used methods, such as Moldflow, DNN, RSM, and NSGA-II, to study the effects of factors, such as the mold temperature, solution temperature, packing pressure, packing time, injection molding time, cooling temperature, and cooling time, on the forming quality of polymer–metal hybrid (PMH) car front panels and obtained the optimal injection molding process parameters to guide actual production. The research results show that the proposed method has great potential in improving injection molding warpage deformation. Mukras et al. [8] aimed to minimize product defects (warpage deformation and volume shrinkage) and conducted actual injection molding experiments based on the face center composite design method. They analyzed the effects of seven process parameters, including the mold temperature, melt temperature, holding pressure, holding time, cooling time, injection speed, and injection pressure, on warpage deformation and volume shrinkage, and used a genetic algorithm to achieve multi-objective optimization. Zhou et al. [9] proposed a differential sensitivity fusion method (DSFM) that integrates a sampling strategy, numerical simulation, meta-modeling method, and a multi-objective optimization algorithm to achieve the multi-objective optimization of injection molding process parameters for automotive front bumpers. Ding et al. [10] proposed a multi-objective optimization method for the injection molding of thin-walled plastic parts based on tuna swarm optimization using a support vector machine (TSO-SVM) and multi-objective sparrow search algorithm (MOSSA), which solves the problems of warping deformation and volume shrinkage during the injection molding process. Nguyen et al. [11] combined orthogonal experiments, response surface methodology (RSM), and the NSGA-II method to optimize the quality process parameters of centrifugal pump casings during injection molding. Cao et al. [12] used simulation, the Latin hypercube sampling method, Bayesian optimized random forest regression (BO-RFR), gradient enhanced regression (GBR), and support vector regression (SVR) to construct a prediction model for process parameters, warpage, and volume shrinkage. Wang et al. [13] proposed a hybrid multi-objective optimization method that combines gradient-enhanced kriging (GEK) with multi-population differential evolution (MPDE) to minimize the warpage, volume shrinkage, and cycle time as optimization parameters. Wu et al. [14] proposed a multi-objective optimization method for the nonlinear shrinkage of micro-injection molded small modulus plastic gears based on a Moldflow simulation combined with a second-order response surface model and non-dominated sorting genetic algorithm II. The research results indicate that the most significant factor affecting the shrinkage of small modulus plastic gears is the holding time, and there are complex interactions between process parameters. Tan et al. [15] combined the Taguchi method with grey relational analysis to study the effects of the melt temperature, mold temperature, injection time, holding pressure/time, and cooling time on the size shrinkage and warping of wire harness connectors. Zeng et al. [16] proposed a multi-objective optimization method for injection molding process parameters based on hierarchical sampling and the comprehensive entropy weight to optimize the injection molding parameters of thin-walled propeller blades. The results showed that it was more optimized in multi-objective optimization than the response surface method. Hentati et al. [17] used the Taguchi method to study the effects of different molding parameters, such as the material temperature, injection pressure, holding time, and mold temperature, on the tensile stress (σ) and Young’s modulus (E) of PC/ABS blends. The research results showed that the injection pressure and material temperature had the most significant impact on the mechanical properties and microstructure of PC/ABS blends. Guo et al. [18] aimed to optimize the production energy consumption, weight, and warpage. Based on Latin hypercube sampling, they established an optimal neural network model to predict the complex relationship between metal injection molding process parameters and the energy consumption, quality, and warpage. They used the NSGA-II algorithm and fuzzy decision-making based on criticism to achieve process parameter optimization. Liu et al. [19] took the warpage, clamping force, and sink marks as optimization objectives, and five process parameters, including the holding pressure, melt temperature, holding time, injection time, and melting time, were analyzed. They used the Taguchi orthogonal experiment, PSO-BP neural network model, improved particle swarm optimization algorithm, and TOPSIS method to achieve multi-objective optimization. Lee et al. [20] used an artificial neural network model to predict the nonlinear relationship between injection molding process parameters, such as the melt temperature, mold temperature, injection speed, holding pressure, holding time, cooling time, and product performance (quality, diameter, and height). Chang et al. [21] used the Pareto optimization framework and injection molding process parameters to perform multi-objective optimization based on the quality of drone shell parts. The research results showed that the injection time and pressure time are positively correlated with the mold indicators, with the strongest correlation, while the mold temperature shows a certain degree of a negative correlation. Li et al. [22] proposed an adaptive optimization method that combines the Design of Experiments (DOE) method with the Kriging surrogate model to reduce the warpage and residual stress of the bracket during injection molding. Yang et al. [23] used Moldflow software, uniformity testing, and the GA-BP neural network model to study the effects of five process parameters, including the mold temperature, melt temperature, holding pressure, injection time, and holding time, on the volume shrinkage rate and warpage variables of automotive plastic front frames. Gianluca et al. [24] adopted a central composite experimental design with flash formation and the component weight as optimization objectives, analyzing five process parameters, including the melting temperature, holding pressure, mold temperature, injection speed, and holding time. Regression equations and utility functions were used to achieve multi-objective optimization. Chen et al. [25] optimized the injection molding process parameters of plastic bottle preform parts based on the GA-BP-PSO algorithm. The research results showed that the warpage deformation obtained using the GA-BP-PSO algorithm differed little from the simulated values of Moldflow software. Li et al. [26] established a Kriging model based on orthogonal experiments and used numerical optimization algorithms, direct search methods, and global exploration methods to optimize the model, to obtain the optimal injection parameters with the minimum warpage deformation.

Based on the above literature, it can be concluded that using artificial neural network models, the NSGA-II algorithm, and the particle swarm optimization algorithm to study the optimization of plastic injection molding process parameters is reliable. To improve the quality and work efficiency of plastic injection molding, this study proposes a multi-objective optimization design method for the key process parameters of the plastic injection molding product quality, which combines the BP neural network and NSGA-II algorithm. The junction box shell is taken as the research object, and orthogonal experimental design and Moldflow simulation software are used for numerical simulation to obtain the original sample data. The parameters that have a significant impact on the quality of plastic parts are determined through variance analysis of the signal-to-noise ratio. Based on the BP neural network and NSGA-II, the injection molding process parameters of junction boxes are optimized to reduce the amount of warping deformation generated during the molding process and improve product quality.

## 2. Establishment of Finite Element Model and Process Analysis

### 2.1. Structural Analysis of Junction Box

**Figure 1:**
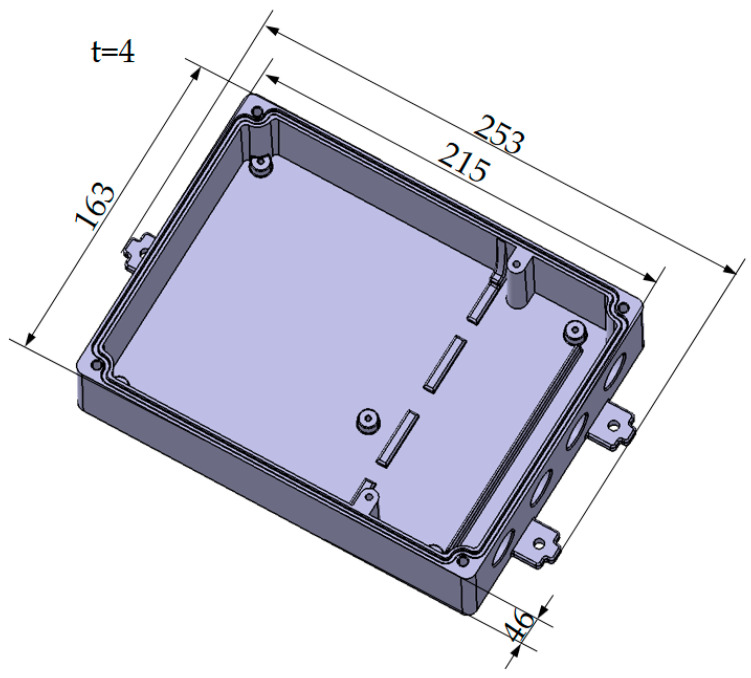
Three-dimensional structural diagram of junction box shell (size unit: mm).

The three-dimensional structure of the junction box housing is shown in Figure 1. As the chart shows, the shape of the junction box housing is irregular, with protrusions and holes. In the process of melt injection molding, it is necessary first to analyze the structure of the plastic parts and adopt reasonable injection methods to obtain higher-quality products. The plastic material is ABS, one of the widely used engineering plastics with excellent molding and mechanical properties. The recommended melt temperature is 200–280 °C, and the mold temperature is 25–80 °C [27].

### 2.2. Analysis of Mold Flow Under Initial Process Parameter Conditions

Grid division determines the accuracy of the discretization of finite element models and plays a crucial role in the accuracy and reliability of finite element analysis results. The neutral layer grid division speed is fast, but it cannot provide high accuracy; the Dual Domain with double-layer mesh is suitable for most product solutions and can provide a more accurate analysis and optimization; 3D solid meshes are often suitable for parts with complex structures and uneven thicknesses. A large number of meshes can lead to reduced efficiency and improved computational accuracy in finite element soft computing. Considering that the shape of the wiring housing is relatively simple and the wall thickness is relatively uniform in the overall structure, a double-layer mesh division method is adopted. Import the 3D model of the junction box housing into Moldflow software for automatic meshing. Pre-process the complex corners, sharp edges, and other structures of the injection molded parts through mesh repair to ensure that there were no multiple edges, free edges, intersecting elements, or completely overlapping elements. The mesh quality used for the simulation calculation is shown in Table 1, indicating that the mesh quality is good and there are no other defects. It is suitable for double-layer surface analysis and can be used for the Moldflow mold flow analysis simulation calculation.

**Figure 2:**
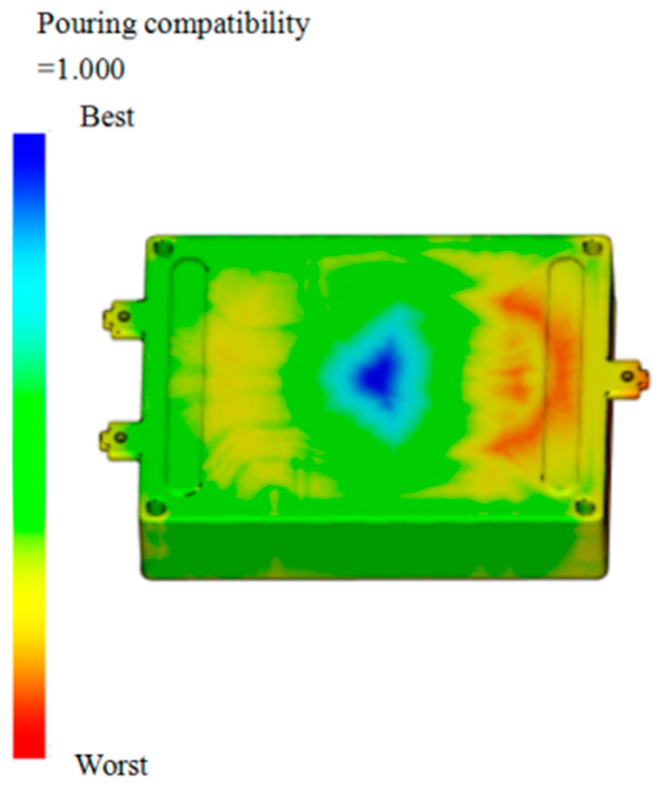
Optimal gate location.

The setting of the gate position of the junction box is related to the flow mode and flow time of the plastic melt in the mold [28]. Determine the optimal gate position for the junction box based on the Moldflow software, as depicted in Figure 2.

According to the optimal gate position of the junction box shown in Figure 2, it can be seen that the blue area at the center of the plastic part has the best melt flowability and is more suitable as the optimal gate position. Considering the simple structure of the junction box plastic parts, to further reduce the number of defects, such as air pockets and weld lines that may be encountered during the analysis process, the final number of gates is determined to be 1.

**Figure 3:**
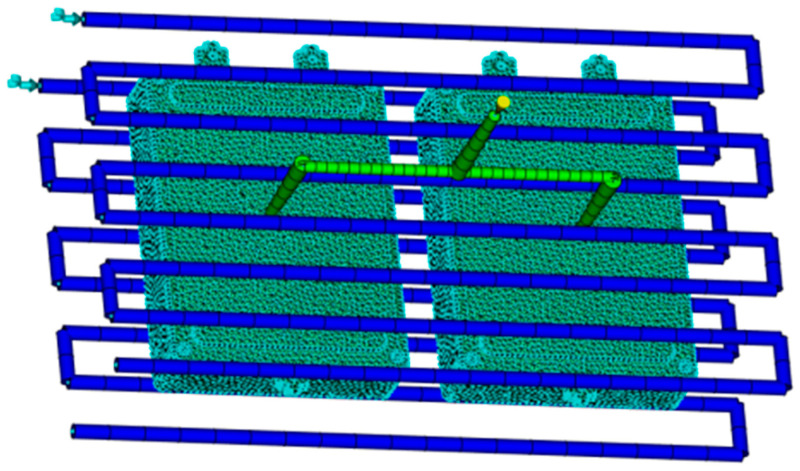
Overall finite element analysis model.

The design of the pouring system has a significant impact on the forming quality of the junction box. Based on practical engineering experience, the first mock examination and two cavity pouring schemes are selected for the pouring system. To ensure the surface quality of the junction box shell, the gate position is fixed on the outside of the product. The cooling system can evenly cool the product after molding, reduce residual stress, maintain the dimensional accuracy of the plastic parts, and improve quality. The cooling system of the junction box adopts a simple and easy-to-process DC circulating cooling water channel, with specific parameters: the diameter of the water pipe is 10 mm, and the distance between the water pipe and the plastic parts of the parts is 20 mm. Establish a finite element model of the junction box for the pouring system and cooling system as shown in Figure 3.

Determine the layout of the pouring and cooling system, conduct mold flow analysis on the junction box, set the analysis window to cooling + filling + holding pressure + warping, and set the process parameters to default values, as follows: the melt temperature is 240 °C, the mold temperature is 52.5 °C, the mold opening time is 5 s, the injection + holding pressure + cooling time is specified as 30 s, filling control is automatic, speed/pressure switching is automatic, the holding pressure control is filling pressure and time, without considering mold thermal expansion and angular effects. The analysis shows that the volume shrinkage rate and warpage deformation of the junction box are 10.35% and 1.352 mm, respectively.

## 3. Orthogonal Experiments

Considering that many factors affect the quality of the injection molding of junction boxes, it is difficult to measure them through process experiments or finite element simulation methods. To reduce computational costs and the number of experiments, the orthogonal experiments were used to optimize the experimental design. To investigate the influence of the injection molding process parameters on the warpage deformation of junction boxes, the melt temperature (A), mold temperature (B), injection pressure (C), holding pressure (D), holding time (E), and cooling time (F) were selected as factors for orthogonal experiments. Each factor was set at five levels to establish an orthogonal table of L25 (5^6^). The experimental factors and factor levels are shown in Table 2.

Using Moldflow simulation software to simulate the six-factor five-level orthogonal experiment described in Table 2, the evaluation indicators are the volume shrinkage rate (X_1_/%) and maximum warpage (X_2_/mm). The results of 25 sets of experiments are as described in Table 3.

Based on the numerical simulation results of the injection molding of the junction box obtained from Table 3, the volume shrinkage rate and maximum warpage deformation degree of the junction box were statistically analyzed, and the average volume shrinkage rate and maximum warpage value of each factor and level were obtained. The signal-to-noise ratio range analysis was conducted on the volume shrinkage rate and warpage deformation of the junction box, and the results are shown in Table 4 and Table 5. K_1_–K_5_ in Table 4 and Table 5 represent the statistical mean values at different levels (1–5) and R represents the range of the statistical mean values. The larger the range, the more significant the impact of this factor on the examined indicators.

In the study, the small size characteristic was used to analyze the difference between the volume shrinkage rate and warpage deformation. The larger the signal-to-noise ratio, the smaller the volume shrinkage rate and deformation obtained. From Table 4 and Table 5, it can be seen that the factors affecting the volume shrinkage rate are A > E > B > C > F > D. The optimal process parameter combination is A_1_B_5_C_5_D_5_E_5_F_5_, while the factors affecting the maximum warpage amount are E > D > B > A > F > C, and the optimal process parameter combination is A_4_B_2_C_5_D_5_E_3_F_4_. By comparing with references [29,30,31], it can be seen that the influence of injection molding process parameters on the volume shrinkage rate and warpage deformation of plastic parts is different, and the process parameters also affect each other. Since ACB is the most significant factor affecting the volume shrinkage rate, A_1_C_5_B_5_ is selected here; ED is the most significant factor affecting the maximum warpage deformation, and E_3_D_5_ is selected. The influence of F on the volume shrinkage rate is greater than the maximum warpage deformation, so F_5_ is selected. Finally, the overall optimal combination is determined to be A_1_B_5_C_5_D_5_E_3_F_5_. By inputting the corresponding parameters into Moldflow software for simulation, a volume shrinkage rate of 7.128% and a maximum warpage deformation of 0.938 mm were obtained.

## 4. BP Neural Network

Due to the complex nonlinear relationship between injection molding process parameters and warpage deformation and the volume shrinkage of junction boxes, a BP neural network can be used to establish a prediction model for warpage deformation and the volume shrinkage of junction boxes under different process parameters. To accurately predict the warpage deformation and volume shrinkage of the junction box during the injection molding process, a three-layer BP neural network prediction model was constructed using six process parameters of injection molding as input variables and the volume shrinkage rate and warpage deformation as output variables. Therefore, the BP neural network has six input layer nodes and two output layer nodes, while the number of hidden layer nodes is mainly obtained through empirical formulas. The expression for a three-layer neural network is as follows [32]:

$${net}_{i}=\sum_{j=1}^{n}{x_{i}\omega }_{ij}-\theta$$

(1) $${\;y}_{i}=f\left({net}_{i}\right)\;$$

In Equation (1), *x_i_* is the input layer variable; *y_i_* is the output layer variable; *ω_ij_* and *θ* represent the weights and thresholds of neurons, respectively.

In the experimental model, the S-type tangent function Tansig is used to verify the neuron activation function of the BP neural network, and the linear function Purelin is used as the neuron activation function in the output layer. The training function of the neural network is “Traingdm”. To make the network converge quickly and solve more conveniently and avoid the phenomenon of neuron output saturation, the experimental data obtained through experiments will be normalized according to Equation (2) so that the data of the model training samples should be within the range of [−1, 1].

(2) $$x=\frac{2\times (x-x_{min})}{x_{max}-x_{min}}-1$$

where *x* is the raw data, *y* is the normalized result of *x*, *x_min_* is the minimum value of the raw data, and *x_max_* is the maximum value of the raw data.

**Figure 4:**
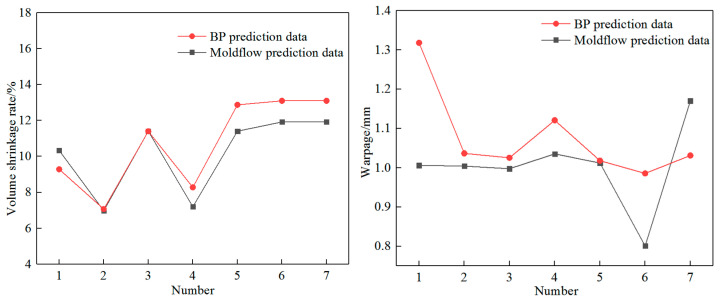
Comparison between BP neural network model predicted values and Moldflow simulation values.

Using 25 sets of orthogonal experimental results as sample data, randomly select the first 18 sets of samples for model training, and use the remaining sample data for model validation. The training objective of the BP neural network model is set to 10^−5^, with a learning rate of 0.1, 100 iterations, and six validation failures. Using the coefficient of determination *R^2^* to determine the degree of fit of the regression equation in the training set, if *R^2^* is closer to 1, the linkage between the predicted and output results is closer, indicating a higher degree of fit of the neural network model. Using MATLAB 2019b software to train the BP neural network prediction model, it was found through trial and error that when the number of hidden layer nodes is five, the 6-5-2-2 three-layer BP neural network model established fits the best *R^2^* = 0.978. At this time, the BP neural network model predicts the volume shrinkage rate and maximum warpage deformation and compares it with the Moldflow simulation values as shown in Figure 4.

According to Figure 4, it can be seen that the established BP neural network model is available to reflect the non-linear relation between the injection molding process parameters of the junction box and its volume shrinkage rate and warpage deformation. This neural network model has certain reliability and can be used for multi-target optimization of the injection molding process parameters of the junction box. Compared with references [19,20], it is feasible to establish an intrinsic mapping relationship between the molding quality and injection molding process parameters using the implicit statistical inference function of multi-layer feedforward neural networks, and the predicted results are relatively accurate. The use of artificial neural network models to achieve intelligent process planning is of great significance for engineering applications.

## 5. Process Parameter Optimization Based on NSGA-II Algorithm

### 5.1. NSGA-II Algorithm

To obtain the optimal injection molding process parameters, the corresponding single objective quality prediction model is often established, and methods, such as the GA algorithm [8], PSO algorithm [33], etc., are commonly used to globally optimize the injection molding process. For the established multi-objective quality prediction model, the multi-objective particle swarm optimization algorithm [9], multi-objective genetic algorithm [34], NSGA-II algorithm [21], etc., are commonly used. The NSGA-II algorithm is an improvement based on the NSGA algorithm, which puts forward a fast and elite non-dominated sorting algorithm. It can not only reduce the computation but also merge the parent population with the child population, allowing the next generation to select from double the space and retain of all the most excellent individuals. The introduction of an elite retention strategy and competitive bidding mechanism ensures that certain excellent individuals in the population will not be discarded during the evolution process, thereby raising the precision of optimization results. By using a crowded-comparison operator, not only does it overcome the defect of manually specifying shared parameters in NSGA, but it also serves as a comparison standard between individuals in the population, allowing individuals in the quasi-Pareto domain to be uniformly extended to the entire Pareto domain, ensuring the diversity of the population. Using the NSGA-II algorithm to tackle the multi-objective problems is beneficial for obtaining the Pareto optimal solution with the minimum deviation between each objective function and the expected value.

### 5.2. Process Parameter Optimization

The nonlinear coupling model between the process parameters of the junction box constructed using the BP neural network and the volume shrinkage rate and warpage is used as the fitness function in the NSGA-II algorithm, and the technology parameters are civilized through the NSGA-II algorithm. The design variables and their ranges are shown below.

**Figure 5:**
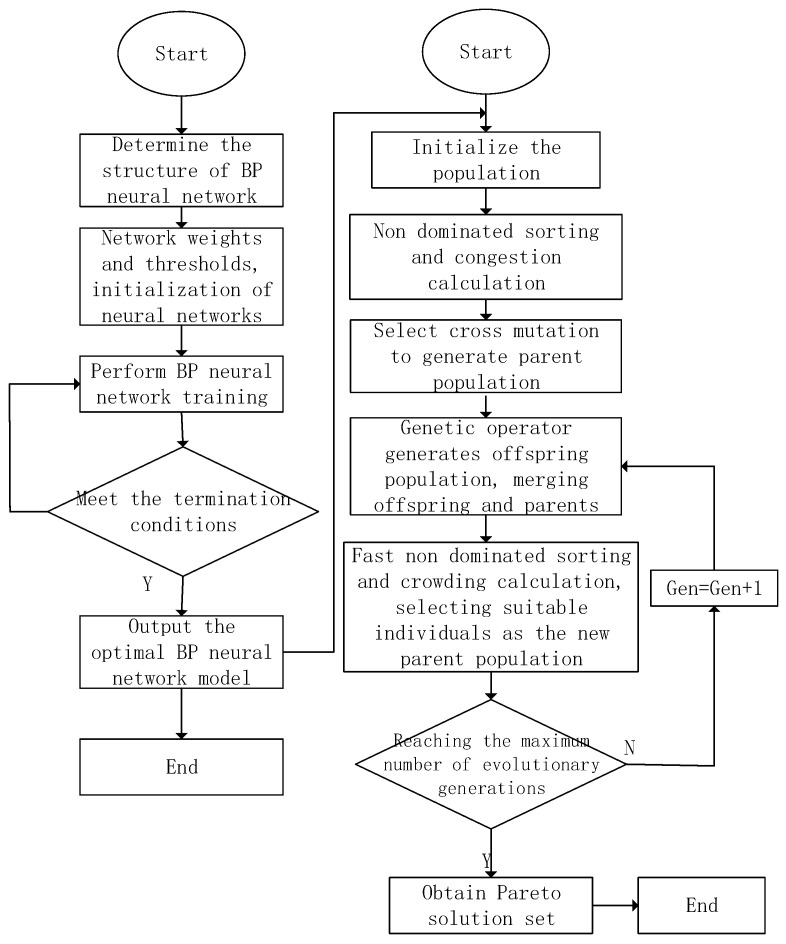
Optimization flowchart of BP neural network combined with NSGA-Ⅱ algorithm.

The melt temperature (A) is 230–270 °C, the mold temperature (B) is 50–90 °C, the injection pressure (C) is 40–60 MPa, the holding pressure (D) is 50–70 MPa, the holding time (E) is 10–30 s, and the cooling time (F) is 15–35 s. The optimization process of the BP neural network combined with the NSGA-II algorithm is shown in Figure 5.

**Figure 6:**
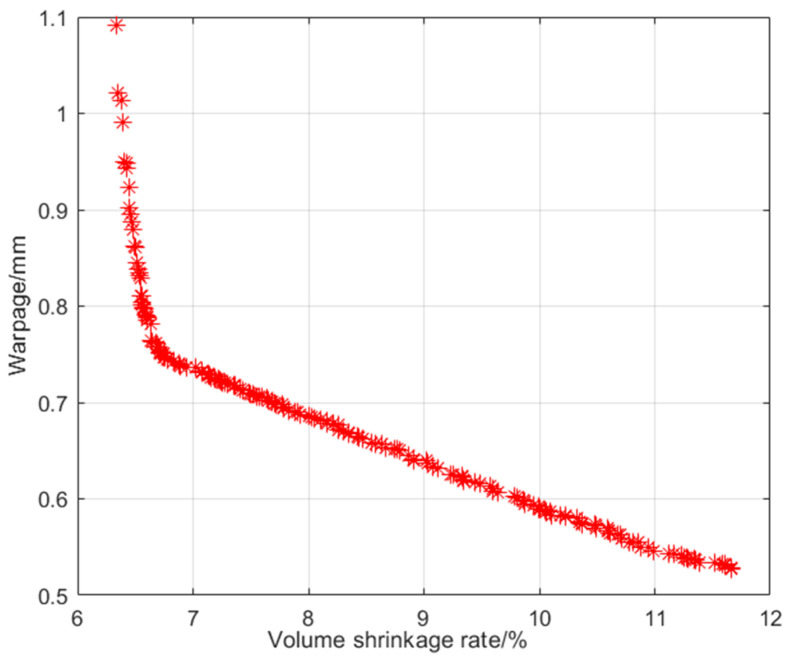
NSGA-Ⅱ optimization results.

When optimizing, the fitted BP neural network model is integrated into the main program of the algorithm in the form of an equation as the source of the initial population. The number of inputs is 6, the number of outputs is 2, the population size is set to 500, the crossover probability is set to 0.8, the mutation probability is set to 0.3, the maximum number of iterations is set to 50, and other parameters are set to default values. After 50 iterations of running the NSGA-II algorithm program in MATLAB, the Pareto solution was obtained as shown in Figure 6.

According to the optimization results shown in Figure 6, the algorithm has good convergence and can obtain a uniformly distributed Pareto optimal solution set. In this study, the multi-objective optimization model has two objective functions, and its Pareto front is a straight line. This indicates that the optimization trends of the volume shrinkage rate and warpage deformation of the junction box are opposite; that is, smaller warpage deformation corresponds to a larger volume shrinkage rate. It is difficult to obtain the optimal process parameters that achieve the minimum of both at the same time. In reference [19], the equal-weight TOPSIS method was used to obtain the optimal solution from the Pareto optimal frontier. In this study, the CRITIC comprehensive evaluation method was used to analyze and obtain the optimal process parameters, and both the volume shrinkage rate and warpage variables were found to be optimal. TOPSIS ranks each solution based on its proximity to the optimal and worst solutions, to determine the optimal design [35]. TOPSIS is suitable for multi-criteria decision-making and ranking problems. The CRITIC weighting method calculates and ranks the weights by constructing a judgment matrix and calculating consistency indicators. The CRITIC weighting method is suitable for multi-criteria evaluation and decision-making problems. The specific expression of the comprehensive evaluation model is as follows [36]:

(3) $$Z=\sum_{j=1}^{2}W_{j}y_{ij}=W_{1}y_{i1}+W_{2}y_{i2}$$

For the 50 sets of solutions obtained through NSGA-II multi-objective optimization, objective weights for the volume shrinkage rate and warpage deformation are calculated based on CRITIC. The general steps are as follows:

(1) Determine the indicator data matrix. There are a total of 50 samples to be evaluated, and the evaluation matrices for two evaluation indicators are as follows:
  (4) $$Y=y_{ij}=\left[\begin{array}{cc} y_{11} & y_{12}\\ \vdots & \vdots \\ y_{50\times 1} & y_{50\times 2} \end{array}\right]$$
  Equation (4), *y_ij_* represents the value of the *jth* evaluation index in the i-th experiment.
(2) Dimensionless processing.
  To remove effects of different dimensions on the assessment, it is necessary to make the volume shrinkage rate and warpage deformation dimensionless. The smaller the indicators to be optimized, the better. Therefore, when standardizing, negative indicators are used for the calculation.
  (5) $$y_{ij}^{\prime }=\frac{\left(y_{j}^{max}-y_{ij}\right)}{y_{j}^{max}-y_{j}^{min}}$$
  Equation (5): $y_{ij}^{\prime }$ represents the standardized value, and *y^max^* and *y^min^* are the upper and lower limits of indicator *j*.
(3) Calculate indicator variability *σ_j_*
  In the CRITIC method, the standard deviation is used to represent the fluctuation of values within each indicator. The larger the standard deviation, the greater the numerical difference of the indicator and the stronger the evaluation strength of the indicator itself. Therefore, more weight should be assigned to the indicator.
  (6) $$\bar{y_{j}}=\frac{1}{n}\sum_{i=1}^{n}y_{ij}^{\prime }$$
  (7) $$\sigma_{j}=\sqrt{\frac{\sum_{i=1}^{n}{{(y}_{ij}^{\prime }-\bar{y})}^{2}}{n-1}}$$
  In Equations (6) and (7), *n* is the total number of experimental groups, and *σ_j_* represents the standard deviation of the jth indicator.
(4) Calculate indicator conflict R_j_
  (8) $$R_{j}=\sum_{i=1}^{n}\left(1-r_{ij}\right)$$
  In Equation (8), *r_ij_* represents the correlation coefficient between evaluation indicators *i* and *j*. The correlation coefficient is used to represent the correlation between indicators. The stronger the correlation with other indicators, the less conflicting the indicator is with other indicators, reflecting more similar information. The evaluation content that can be reflected is more repetitive, which weakens the evaluation strength of the indicator to a certain extent. Therefore, the weight assigned to the indicator should be reduced.
(5) Calculate the amount of information C_j_
  (9) $$C_{j}=\sigma_{j}\times R_{j}$$
  The larger the *C_j_*, the greater the role of the jth evaluation indicator in the entire evaluation indicator system, and it should be distributed with more weight.
(6) Calculate objective weight *W_j_*
  (10) $$W_{j}=\frac{C_{j}}{\sum_{j=1}^{n}C_{j}}$$
  According to Equations (5)–(10) above, gradually calculate the objective weight values of the two indicators. As shown in Table 6, the objective weight of the volume shrinkage rate in the two molding masses is 0.4878, and the objective weight of warpage deformation in the two molding masses is 0.5122. Based on the obtained weight values, calculate the comprehensive evaluation value of the molding quality for each group of schemes according to Equation (1). Among them, the comprehensive evaluation value of the injection molding process scheme corresponding to a volume shrinkage rate of 6.905% and a warpage deformation of 0.991 mm reached 3.876, which is the minimum value among the 50 schemes. At this time, the optimal process parameter combination is the melt temperature of 230.03 °C, mold temperature of 51.27 °C, injection pressure of 49.13 MPa, holding pressure of 69.01 MPa, holding time of 15.48 s, and cooling time of 34.91 s.

**Figure 7:**
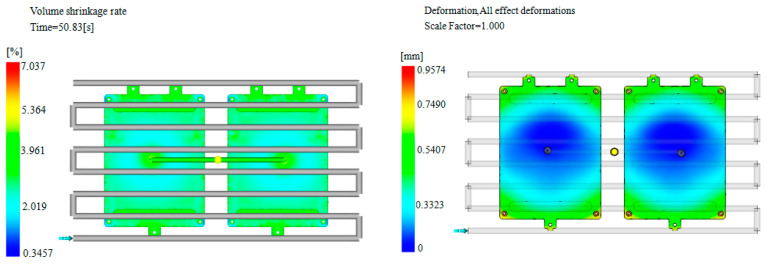
Numerical simulation results after algorithm optimization.

Comparing the optimal results of the process parameters before optimization in Table 3, it can be seen that the NSGA-II algorithm improves the warpage deformation during the injection molding process of the junction box. The above process combination was applied to Moldflow for the numerical simulation calculation, and the calculated volume shrinkage rate was 7.037%, with a warpage deformation of 0.957 mm, as shown in Figure 7. The relative errors of the volume shrinkage rate and warpage deformation predicted by the NSGA-II algorithm were 1.9% and 3.4%, respectively, indicating a high degree of agreement between the two. This is similar to the findings of the literature [11,18,19,35]. By establishing a multi-objective optimization model and using the NSGA-II algorithm, the injection molding process parameters can be optimized to improve the molding quality of injection molded products.

## 6. Conclusions

(1) Based on a six-factor five-level orthogonal experiment, the influence of the melt temperature, mold temperature, injection pressure, holding pressure, holding time, and cooling time on the volume shrinkage rate and warpage deformation of junction boxes was studied using Moldflow simulation software. By analyzing the range of the signal-to-noise ratio, the primary and secondary order of the influence of injection molding process parameters on the volume shrinkage rate and warpage deformation of junction boxes is obtained as follows: melt temperature > holding time > holding pressure > cooling time > injection pressure > mold temperature.
(2) Based on a fitted BP neural network model and combined with the NSGA-II algorithm, multi-target optimization was carried out within a specified parameter range using the quality indicators to determine the optimal solution, with a melt temperature of 230.03 °C, mold temperature of 51.27 °C, injection pressure of 49.13 MPa, holding pressure of 69.01 MPa, holding time of 15.48 s, and cooling time of 34.91 s. The resulting product had a warpage deformation of 0.912 mm, which was 33.2% lower than the average volume fraction before optimization (10.34884%). The optimal results were significant, and the injection molding quality of the junction box was improved.
(3) The errors between the volume shrinkage rate and warpage deformation predicted using BP-NSGA-II and the simulated values using Moldflow software are 1.9% and 3.4%, respectively, indicating that the optimization method of the BP neural network and NSGA-II algorithm is reliable.

## Figures and Tables

**Table 1:** Grid quality of junction box shell.

Item	Actually	Requirement
Mesh-type	Triangles	—
Maximum aspect ratio	16.0	<20
Average aspect ratio	2.96	<3
Fully overlapping elements	0	0
Match percentage	86%	>85%
Total grids	51,388	—

**Table 2:** Factors and levels of orthogonal experiments.

	Factor	Melt Temperature/°C	Mold Temperature/°C	Injection Pressure/MPa	Holding Pressure/MPa	Holding Time/s	Cooling Time/s
Level							
1		230	50	40	50	10	15
2		240	60	45	55	15	20
3		250	70	50	60	20	25
4		260	80	55	65	25	30
5		270	90	60	70	30	35

**Table 3:** Results of orthogonal experiment.

Number	Experimental Factors						Experimental Result	
	A/℃	B/℃	C/MPa	D/MPa	E/s	F/s	X_1_/%	X_2_/mm
1	1	1	1	1	1	1	7.661	1.307
2	1	2	2	2	2	2	7.206	1.035
3	1	3	3	3	3	3	7.095	1.022
4	1	4	4	4	4	4	7.018	1.015
5	1	5	5	5	5	5	6.981	1.004
6	2	1	2	3	4	5	10.35	1.018
7	2	2	3	4	5	1	10.35	1.023
8	2	3	4	5	1	2	10.35	1.106
9	2	4	5	1	2	3	10.34	1.056
10	2	5	1	2	3	4	10.34	1.006
11	3	1	3	5	2	4	10.87	0.8403
12	3	2	4	1	3	5	10.87	1.008
13	3	3	5	2	4	1	10.87	1.015
14	3	4	1	3	5	2	10.88	1.016
15	3	5	2	4	1	3	10.88	1.281
16	4	1	4	2	5	3	11.41	1.013
17	4	2	5	3	1	4	11.42	1.012
18	4	3	1	4	2	5	11.41	0.9905
19	4	4	2	5	3	1	11.41	0.8618
20	4	5	3	1	4	2	11.41	0.9973
21	5	1	5	4	3	2	11.92	0.8007
22	5	2	1	5	4	3	11.92	0.8195
23	5	3	2	1	5	4	11.92	1.01
24	5	4	3	2	1	5	11.92	1.342
25	5	5	4	3	2	1	11.92	1.17

**Table 4:** Analysis of volume shrinkage ratio signal-to-noise ratio.

Mean Signal-to-Noise Ratio	Experimental Factors					
	A	B	C	D	E	F
*K*_1_	−17.13	−20.28	−20.28	−20.27	−20.28	−20.28
*K*_2_	−20.30	−20.17	−20.17	−20.17	−20.17	−20.17
*K*_3_	−20.73	−20.14	−20.14	−20.15	−20.14	−20.14
*K*_4_	−21.15	−20.12	−20.12	−20.13	−20.12	−20.12
*K*_5_	−21.53	−20.11	−20.11	−20.11	−20.12	−20.11
*R*	4.39	0.16	0.16	0.16	0.16	0.16
Ranking	1	3	4	6	2	5

**Table 5:** Analysis of warpage ratio signal-to-noise ratio.

Mean Signal-to-Noise Ratio	Experimental Factors					
	A	B	C	D	E	F
*K*_1_	−0.59547	0.16985	−0.14068	−0.58619	−1.60205	−0.54484
*K*_3_	−0.19522	−0.23939	−0.28136	−0.38984	0.58246	−0.23929
*K*_4_	0.23649	−0.40073	−0.50991	−0.09286	0.26778	0.22800
*K*_5_	−0.06911	−0.71557	0.23794	0.72449	−0.11374	−0.54620
*R*	0.83196	0.92755	0.74785	1.35395	2.18451	0.77419
Ranking	4	3	6	2	1	5

**Table 6:** Calculation of objective weights for evaluation indicators.

Indicator	Variability	Conflict-Oriented	Information Content	Weight
volume shrinkage rate	0.255	1.960	0.500	48.78%
warpage	0.268	1.960	0.525	51.22%

## Data Availability

All data, models, and code generated or used during the study appear in the submitted article.
